# Genetically predicted 91 circulating inflammatory proteins in relation to risk of urological malignancies: a Mendelian randomization study

**DOI:** 10.18632/aging.205934

**Published:** 2024-06-13

**Authors:** Jianxiong Xu, Ru Chen, Yucheng Yang, Sufang Xu, Lijing Yao

**Affiliations:** 1 Department of Radiotherapy, The First Hospital of Putian City, Putian, Fujian 351100, China; 2 Department of Proctology of Traditional Chinese Medicine, The First Hospital of Putian City, Putian, Fujian 351100, China

**Keywords:** urological malignancies, kidney cancer, bladder cancer, prostate cancer, inflammatory proteins, Mendelian randomization

## Abstract

Background: Urological malignancies, including kidney, bladder, and prostate cancer, are major health concerns worldwide. Inflammation has been implicated in the pathogenesis of these cancers, and circulating inflammatory proteins may play a role in their development. However, the causal relationship between specific plasma proteins and urological malignancies remains unclear.

Methods: We performed a two-sample Mendelian randomization (MR) analysis using summary statistics from genome-wide association studies (GWAS). Instrumental variables representing genetic variants associated with circulating inflammatory proteins were used to infer causality on the risk of kidney, bladder, and prostate cancer. Four MR methods were utilized to provide robust effect estimates.

Results: Our analysis identified several plasma proteins associated with a lower risk of kidney and bladder cancer, including Eukaryotic translation initiation factor 4E-binding protein 1, Caspase 8, Natural killer cell receptor 2B4, and Tumor necrosis factor ligand superfamily member 12. However, after adjusting for multiple testing, these associations did not remain statistically significant. For prostate cancer, CUB domain-containing protein 1 and Interleukin-10 receptor subunit beta were found to be protective, while Glial cell line-derived neurotrophic factor and SIR2-like protein 2 were identified as risk factors. After FDR adjustment, none of the inflammatory proteins were found to be significantly associated with a lower risk of prostate cancer.

Conclusion: Our findings suggest that certain plasma proteins may be involved in the development of urological malignancies. Mendelian randomization provides a useful framework for investigating causal relationships between inflammatory proteins and urological cancers, offering potential insights into their underlying biology and therapeutic targets.

## INTRODUCTION

Cancer is a leading cause of mortality worldwide, and urological tumors, including kidney, prostate, and bladder cancers, contribute significantly to this burden [1, 2]. Traditionally, the development and progression of cancer have been attributed to genetic mutations and environmental factors. However, emerging evidence now suggests that chronic inflammation also plays a crucial role in the pathogenesis of various cancers. While chronic inflammation is directly associated with cancer development in only about 20% of cases, it is believed to indirectly contribute to tumorigenesis in a more substantial percentage of patients [1]. In the context of urological cancers, chronic inflammation induced by bacterial or viral infections is not a primary cause. Instead, other factors like obesity, tobacco smoking, alcohol consumption, and infection can lead to chronic inflammation and subsequently contribute to the development and progression of urological cancers [3, 4].

Obesity, in particular, has been strongly associated with the development of kidney and prostate cancers [5, 6]. Recent studies have shown that obesity promotes the infiltration of macrophages into the prostate tumor microenvironment through specific pathways. In addition to external factors, cancer cells themselves can also trigger inflammation by recruiting and activating inflammatory cells in the tumor microenvironment [7]. In the field of urological cancer research, numerous studies have demonstrated a correlation between the infiltration of inflammatory cells such as neutrophils and macrophages, tumor aggressiveness, and poor prognosis. Neutrophil infiltration, for instance, has been identified as an independent prognostic factor in localized clear cell renal cell carcinoma (ccRCC) [8]. Furthermore, neutrophils have been found to be a significant source of angiogenesis-inducing enzymes in the prostate tumor microenvironment [9].

Cancer-related inflammation is now recognized as the seventh hallmark of cancer, with inflammatory cells and mediators playing a crucial role in the tumor microenvironment [10, 11]. The systemic inflammatory response (SIR), measured through circulating inflammatory markers such as specific serum proteins and blood cell counts, has been associated with survival in various cancer types. These markers, including C-reactive protein, the Glasgow Prognostic Score, neutrophil-to-lymphocyte ratio, platelet-to-lymphocyte ratio, and prognostic nutrition index, have been extensively studied for their prognostic and predictive value in urological cancers [12, 13].

With the introduction of molecular-targeted drugs and immune checkpoint inhibitors as treatment options for urological cancers, there has been an increasing interest in evaluating the role of SIR markers in predicting treatment response and patient outcomes. In this study, a comprehensive two-sample Mendelian randomization (MR) analysis was conducted to investigate the causal relationship between 91 circulating inflammatory proteins and three urological cancers, namely kidney cancer (KCa), bladder cancer (BCa), and prostate cancer (PCa).

## MATERIALS AND METHODS

### Study design

**Figure 1:**
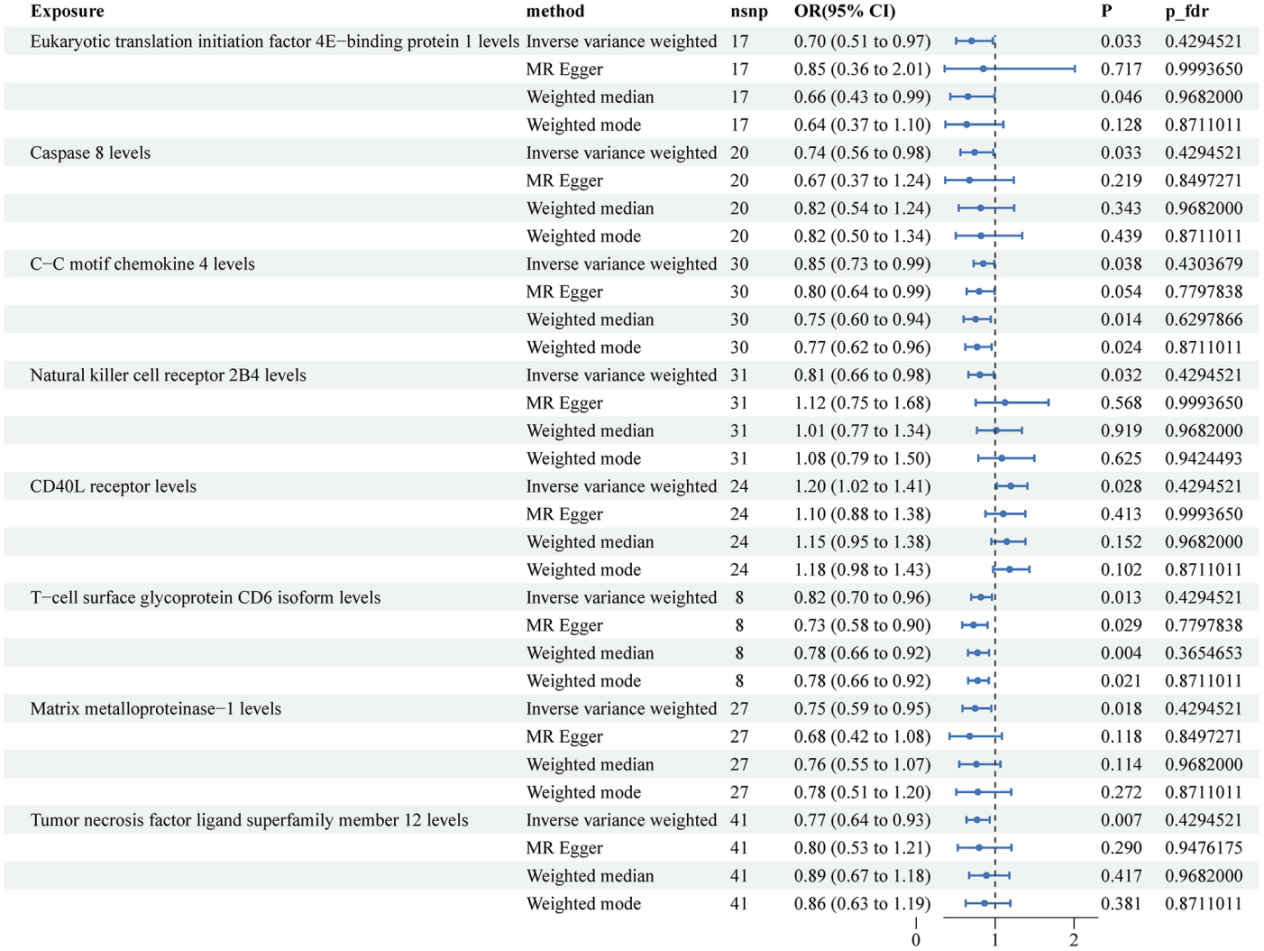
**Assumptions of the Mendelian randomization (MR) analysis for circulating inflammatory proteins and PCa.** The MR study assumes that genetic variants are associated with only circulating inflammatory proteins and not with confounders or alternative causal pathways, that is, the IVs affect the PCa only directly through immune cell signatures. Abbreviation: IVs; instrument variables.

Figure 1 presented the study design and assumptions of Mendelian randomization (MR) used in our study. In this study, we employed summary statistics from genome-wide association studies (GWAS) to conduct MR within a two-sample MR framework. MR analysis is a genetics-based method that uses genetic variation randomly inherited at conception to estimate causal relationships between exposures and outcomes. This approach relies on three critical assumptions: (1) instrumental variables, representing the genetic variants, were strongly associated with the exposure; (2) these genetic variants were not associated with any confounding factors; and (3) there was no direct link between the genetic variants and the outcome (Figure 1). Ethical approval was not required for this study as it relied on publicly available summary data from large-scale GWAS and consortia.

### GWAS data sources for 91 circulating inflammatory proteins and instrument selection

The GWAS Catalog provides publicly available GWAS summary statistics for each circulating inflammatory protein (accession numbers ranging from GCST90274758 to GCST90274848). In this study, genome-wide pQTL mapping was conducted for 91 circulating inflammatory proteins measured using the Olink Target Inflammation panel. A total of 14,824 participants of European ancestry from 11 cohorts were included, and the results were meta-analyzed. According to recent research, a significance level of 1 × 10^−5^ was set for each independent variable (IV) in relation to circulating inflammatory protein. To remove SNPs with a linkage disequilibrium (LD) r2 threshold of <0.001 within a 10,000 kb distance, we utilized the clumping procedure in the PLINK software. To ensure strong instruments and avoid weak instrumental bias, we calculated the proportion of phenotypic variation explained (PVE) and F statistic for each IV. IVs with F statistics below 10 were subsequently removed.

### GWAS data sources for urological malignancies

GWAS summary statistics for prostate cancer were obtained from the Prostate Cancer Association Group to Investigate Cancer Associated Alterations in the Genome (PRACTICAL) consortium [14]. The consortium conducted a GWAS on a population comprising 150,064 individuals of European ancestry, including 79,148 cases and 61,106 controls. Following the application of quality control procedures and imputation, a total of 20,346,368 genetic variants were analyzed. We obtained GWAS summary statistics for bladder cancer from a recent study [15]. This study utilized the fastGWA-GLMM method and analyzed data from the UK Biobank, including 456,348 individuals, 11,842,647 variants, and 2,989 binary traits. The full summary statistics can be accessed at http://fastgwa.info/ukbimpbin. The analysis identified 259 rare variants that are associated with 75 different traits. This study demonstrates the utility of using imputed genotype data in a large cohort to discover rare variants for complex binary traits. GWAS summary statistics for kidney cancer were obtained from a recent study that extensively investigated heritability and pleiotropy in 18 different types of cancer [16]. This study focused on two large, population-based cohorts: the UK Biobank and the Kaiser Permanente Genetic Epidemiology Research on Adult Health and Aging cohorts. The UK Biobank consisted of 408,786 individuals of European ancestry, including 48,961 cancer cases, while the Kaiser Permanente cohort included 66,526 individuals of European ancestry, with 16,001 cancer cases.

### Statistical analysis

Two sample MR was performed to examine the causal relationship between 91 plasma proteins and three type of urological cancers. Instrumental single-nucleotide polymorphisms (SNPs) associated with the exposure were selected from genome-wide association studies (GWASs) on PCa outcomes. To ensure accurate allele harmonization, the SNPs were arranged so that the effect variants for both exposure and outcome matched the same allele. Four MR methods were utilized: inverse variance weighted (IVW), weighted median, MR-Egger, and weighted mode. IVW was used to generate unbiased effect estimates as the main outcome, with the assumption that there was no horizontal pleiotropy. MR-Egger regression analysis was employed to detect and correct for directional pleiotropy, at the expense of reduced power. The *P*-value of the MR-Egger intercept was used to assess directional pleiotropy. The penalized weighted median approach was used to provide consistent effect estimates, assuming that pleiotropic SNPs accounted for less than 50% of MR effect estimates. The weights in this approach were determined by the association strength with the exposure. The weighted median model could yield consistent estimates if more than 50% of the weight came from valid instrumental variables (IVs). Several sensitivity analyses were conducted to ensure the reliability of the MR test, including the MR-Egger intercept test, Cochran’s *Q*-test, and leave-one-out analysis. The MR-Egger intercept analysis was used to assess directional heterogeneity, with a significant deviation from zero indicating its presence. Cochran’s Q statistic was used to identify heterogeneity, with a significant *p*-value suggesting its presence and the subsequent use of the random effects IVW MR method. Leave-one-out analysis helped identify individual SNPs with a disproportionately large effect on the estimates. To account for multiple testing, False Discovery Rate (FDR) correction was applied, with correlations having a *p*-value less than 0.05 considered significant and unadjusted *p*-values between 0.05 and 0.10 considered suggestive. All statistical analyses were performed using the Mendelian Randomization package (version 0.4.2) and Two Sample MR package (version 0.5.5).

### Availability of data and materials

We obtained data from https://gwas.mrcieu.ac.uk/. The original contributions presented in the study are included in the article/Supplementary Material, further inquiries can be directed to the corresponding author.

## RESULTS

Detailed information on genetic instruments of 91 plasma proteins which were used to evaluate effect on KCa, BCa, and PCa were recorded, respectively (Supplementary Tables 1–3). All TL-associated SNPs used as IVs had F-statistics above 10, indicating strong predictiveness of TL and low evidence of weak IVs bias in our study.

**Figure 2:**
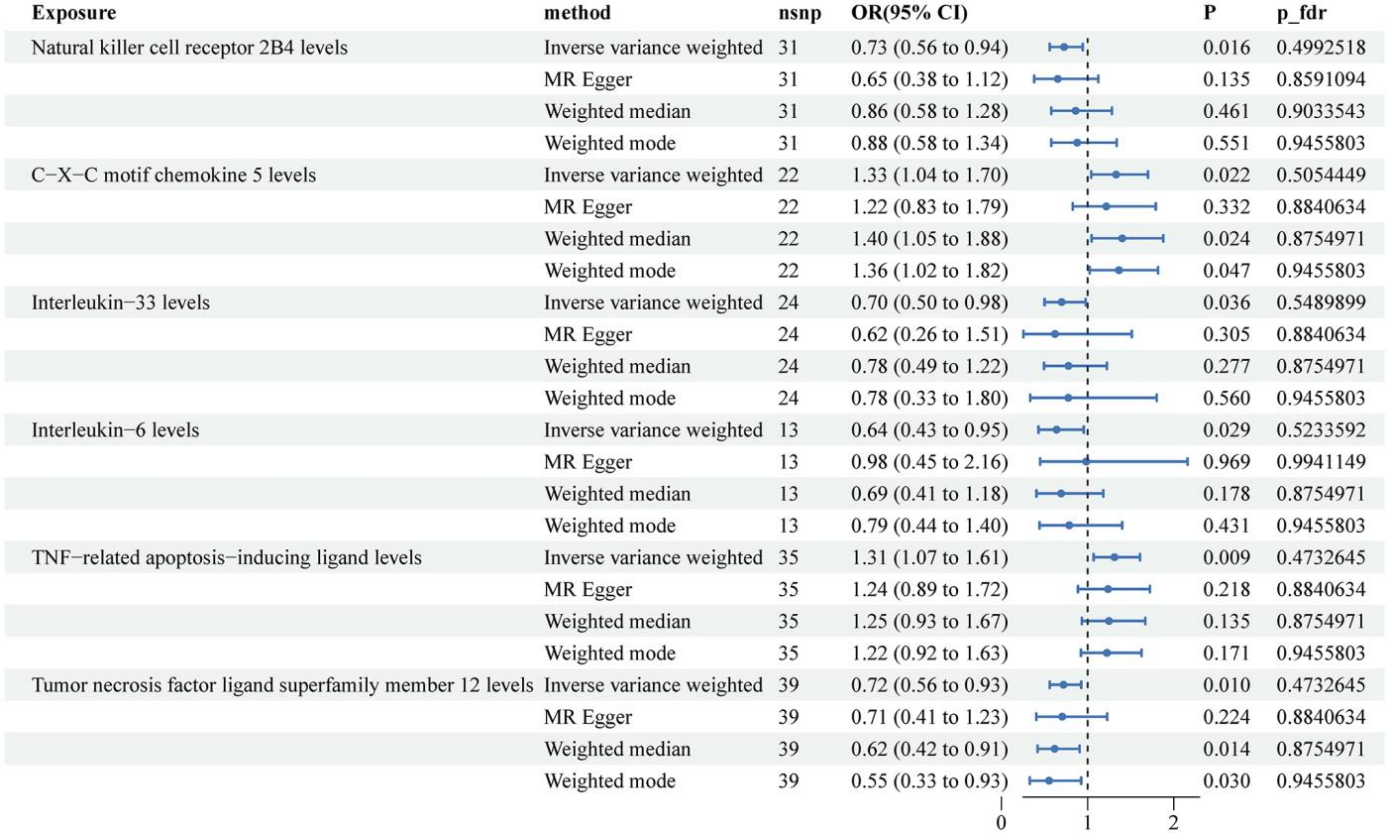
**Associations of genetically predicted circulating inflammatory proteins and the risk of kidney cancer.** Abbreviations: SNPs: single nucleotide polymorphisms; IVW: inverse variance weighted.

The MR IVW results detected 7 plasma proteins associated with a lower risk of KCa: Eukaryotic translation initiation factor 4E-binding protein 1 (OR: 0.70, 95% CI: 0.51–0.97, *P* = 0.033), Caspase 8 (OR: 0.74, 95% CI: 0.56–0.98, *P* = 0.033), C-C motif chemokine 4 (OR: 0.85, 95% CI: 0.73–0.99, *P* = 0.038), Natural killer cell receptor 2B4 (OR: 0.81, 95% CI: 0.66–0.98, *P* = 0.032), T-cell surface glycoprotein CD6 isoform (OR: 0.82, 95% CI: 0.70–0.96, *P* = 0.013), Matrix metalloproteinase-1 (OR: 0.75, 95% CI: 0.59–0.95, *P* = 0.018), and Tumor necrosis factor ligand superfamily member 12 (OR: 0.77, 0.64–0.93, *P* = 0.007) (Figure 2). The CD40L receptor was found to be associated with a higher risk of KCa (OR: 1.20, 95% CI: 1.02 to 1.41, *P* = 0.028). The results of the other three MR methods, namely MR Egger, Weighted Median, and Weighted Mode, were inconsistent with IVW. After adjusting for FDR with a threshold of PFDR <0.05, there were no statistically significant associations observed between these plasma proteins and KCa. The scatter plots for the MR plasma proteins -to-KCa association were presented in Supplementary Figure 1.

**Figure 3:**
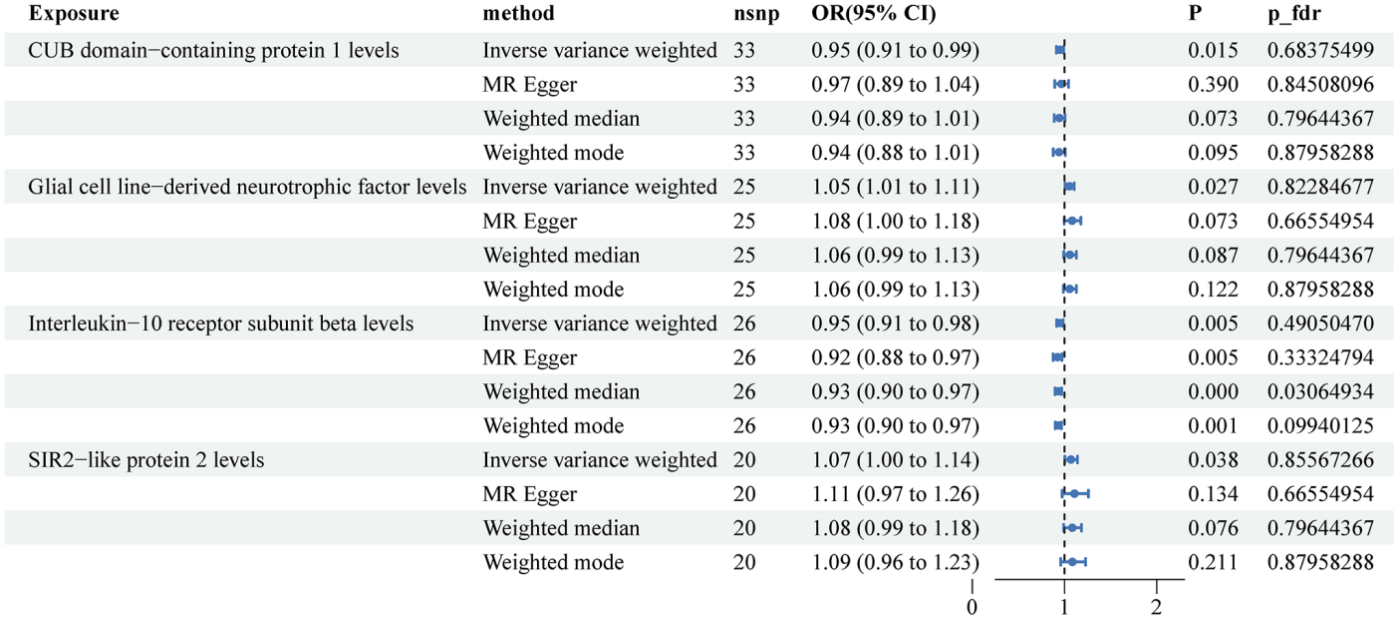
**Associations of genetically predicted circulating inflammatory proteins and the risk of bladder cancer.** Abbreviations: SNPs: single nucleotide polymorphisms; IVW: inverse variance weighted.

Figure 3 displayed the MR results for the association between plasma proteins and BCa. The MR IVW analysis identified four protective factors: Natural killer cell receptor 2B4 (OR: 0.73, 95% CI: 0.56–0.94, *P* = 0.016), Interleukin-33 (OR: 0.70, 95% CI: 0.50–0.98, *P* = 0.036), Interleukin-6 (OR: 0.64, 95% CI: 0.43–0.95, *P* = 0.029), and Tumor necrosis factor ligand superfamily member 12 (OR: 0.72, 95% CI: 0.56–0.93, *P* = 0.01). Additionally, two risk factors were identified: C-X-C motif chemokine 5 (OR: 1.33, 95% CI: 1.04–1.70, *P* = 0.022) and TNF-related apoptosis-inducing ligand (OR: 1.31, 95% CI: 1.07–1.61, *P* = 0.009). However, none of these associations remained statistically significant after adjusting for FDR (all *P* > 0.05). The scatter plots illustrating the associations between plasma proteins and BCa can be found in Supplementary Figure 2.

**Figure 4:**
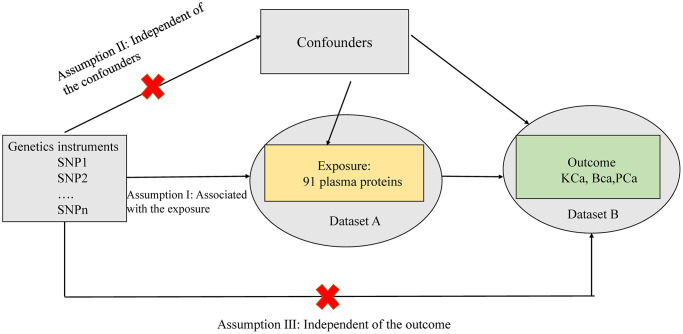
**Associations of genetically predicted circulating inflammatory proteins and the risk of prostate cancer.** Abbreviations: SNPs: single nucleotide polymorphisms; IVW: inverse variance weighted.

The MR results using the IVW method showed two factors that were protective against prostate cancer: CUB domain-containing protein 1 (OR: 0.95, 95% CI: 0.91–0.99, *P* = 0.015) and Interleukin-10 receptor subunit beta (OR: 0.95, 95% CI: 0.91–0.98, *P* = 0.005). Additionally, two factors were identified as risk factors for PCa: Glial cell line-derived neurotrophic factor (OR: 1.05, 95% CI: 1.01–1.11, *P* = 0.027) and SIR2-like protein 2 (OR: 1.07, 95% CI: 1.00–1.14, *P* = 0.038) (Figure 4). The results obtained from the other three MR methods did not align with the IVW results completely. However, after adjusting for the false discovery rate, the Weighted median method showed that Interleukin-10 receptor subunit beta was associated with a lower risk of prostate cancer (OR: 0.93, 95% CI: 0.90–0.97, *P* < 0.001, PFDR = 0.03). The Weighted mode method suggested a potential causal association between Interleukin-10 receptor subunit beta and prostate cancer (OR: 0.93, 95% CI: 0.90–0.97, *P* = 0.001, PFDR = 0.09). Supplementary Figure 3 displays scatter plots illustrating the associations between plasma proteins and prostate cancer in the MR analysis.

Most MR analysis results exhibit heterogeneity (P for Cochrane’s *Q* < 0.05) (Supplementary Table 4). The analysis of the MR-Egger intercept revealed evidence of horizontal pleiotropy in a portion of the MR analysis (*P* < 0.05), suggesting that these findings may not be reliable (Supplementary Table 5). The leave-one-out sensitivity analysis indicated that the genetic prediction of estimating the significant association between plasma proteins and outcomes was robust (Supplementary Figures 4–6).

## DISCUSSION

In this study, we employed a MR study design and assumptions to assess the causal correlation between circulating inflammatory proteins and malignancies in the urogenital system. Our investigation relied on summary statistics derived from GWAS within a two-sample MR framework. The outcomes indicate that several plasma proteins display an association with the risk of urogenital malignancies, although none retained statistical significance after adjusting for confounders.

Emerging evidence indicates that chronic inflammation may have a significant role in the development and progression of most cancers [1, 2]. However, chronic inflammation induced by bacteria or viruses is not typically a direct factor in the development of urological cancers. Nonetheless, certain risk factors like infection, obesity, tobacco smoking, and alcohol consumption, known to contribute to cancer development, may do so through chronic inflammation. Obesity, in particular, has been linked to the development of kidney and prostate cancers [3]. A recent *in vitro* study on prostate cancer demonstrated that obesity promotes the infiltration of macrophages into the tumor microenvironment. This, in turn, leads to the polarization of tumor-associated macrophages via the COX-2/PGE2 pathway [17]. Furthermore, cancer cells themselves can induce inflammation by recruiting and activating inflammatory cells in the tumor microenvironment. These findings highlight the intricate relationship between chronic inflammation and cancer development. While certain risk factors might contribute to inflammation, increasing cancer risk, cancer cells themselves can also initiate inflammation within the tumor microenvironment [7, 17]. In the field of urological cancer research, several studies have demonstrated a strong connection between the presence of inflammatory cells, specifically neutrophils and macrophages, and the aggressiveness of tumors, as well as a poor prognosis [18, 19]. Jensen et al. in a separate study reported that the presence of neutrophils within the tumor is a prognostic factor for shorter periods of recurrence-free, cancer-specific, and overall survival in localized ccRCC [8]. Additionally, Deryugina et al. discovered in a mouse model that neutrophils infiltrating the tissue are a significant source of MMP-9, which is a factor that induces angiogenesis in the tumor microenvironment [9]. Cancer-related inflammation is widely recognized as the seventh hallmark of cancer, with inflammatory cells and molecules such as cytokines and chemokines playing a crucial role in the tumor microenvironment. An essential point to note is that this inflammatory response can be measured in the peripheral blood, and significant associations have been observed between systemic inflammation and survival outcomes in patients with various types of cancer.

Our results revealed potential protective factors for KCa, BCa, and PCa. For KCa, we identified seven plasma proteins, including Eukaryotic translation initiation factor 4E-binding protein 1, Caspase 8, C-C motif chemokine 4, Natural killer cell receptor 2B4, T-cell surface glycoprotein CD6 isoform, Matrix metalloproteinase-1, and Tumor necrosis factor ligand superfamily member 12, that were associated with a lower risk of the disease. However, none of these associations remained statistically significant after adjusting for FDR. For BCa, we found four protective factors: Natural killer cell receptor 2B4, Interleukin-33, Interleukin-6, and Tumor necrosis factor ligand superfamily member 12, as well as two risk factors: C-X-C motif chemokine 5 and TNF-related apoptosis-inducing ligand. Similar to KCa, these associations did not survive FDR correction. Lastly, for PCa, we identified two protective factors: CUB domain-containing protein 1 and Interleukin-10 receptor subunit beta, and two risk factors: Glial cell line-derived neurotrophic factor and SIR2-like protein 2. After FDR correction, none of the inflammatory proteins were found to be significantly associated with a lower risk of PCa.

One of the strengths of our study is the use of summary statistics from GWAS for both the exposure (circulating inflammatory proteins) and the outcomes (urological cancers). This two-sample MR design allows for robust causal inference by leveraging genetic variants as instrumental variables. Furthermore, we employed multiple MR methods, including the IVW, weighted median, MR-Egger, and weighted mode, to ensure the reliability of our findings. It is important to acknowledge the limitations of our study. First, MR analyses rely on certain assumptions, including the instrumental variable assumptions and the absence of horizontal pleiotropy. While we attempted to address these concerns using sensitivity analyses, the presence of unmeasured confounding or pleiotropy cannot be completely ruled out. Second, our study only included participants of European ancestry, which may limit the generalizability of our findings to other populations. Third, the circulating inflammatory proteins examined in this study were measured using the Olink Target Inflammation panel, and the selection of proteins might not fully represent the entire inflammatory pathway. Additionally, the use of GWAS summary statistics may introduce bias if the included studies have different data quality or population characteristics. Lastly, after applying FDR correction, no statistically significant causal relationship was observed between circulating inflammatory proteins and urological cancers. This outcome may be attributed to the stringent criteria we employed in our analysis, which could have led to false-negative results.

## CONCLUSION

Our MR study investigated the potential causal relationship between 91 circulating inflammatory proteins and three types of urological malignancies. We found several plasma proteins that were associated with a lower risk of KCa, BCa, and PCa. However, after adjusting for multiple testing, none of these associations remained statistically significant. Our findings suggest that these circulating inflammatory proteins may not have a direct causal effect on the development of urological malignancies. Further research is needed to validate these results and explore other potential mechanisms underlying the association between inflammatory proteins and urological cancer risk. These findings have important implications for understanding the role of inflammation in urological malignancies and may guide future therapeutic interventions targeting inflammatory pathways.

## Supplementary Materials

Supplementary Figures

Supplementary Table 1

Supplementary Table 2

Supplementary Table 3

Supplementary Table 4

Supplementary Table 5
