# Supplementary material for: Genetically predicted 91 circulating inflammatory proteins in relation to risk of urological malignancies: a Mendelian randomization study
Source: Aging (Albany NY). 2024 Jun 13;16(12):10366–79. doi: 10.18632/aging.205934 (PMC11236305; doi:10.18632/aging.205934)
Supplement: Supplementary Figures [file aging-16-205934-s001.pdf]

SUPPLEMENTARY FIGURES

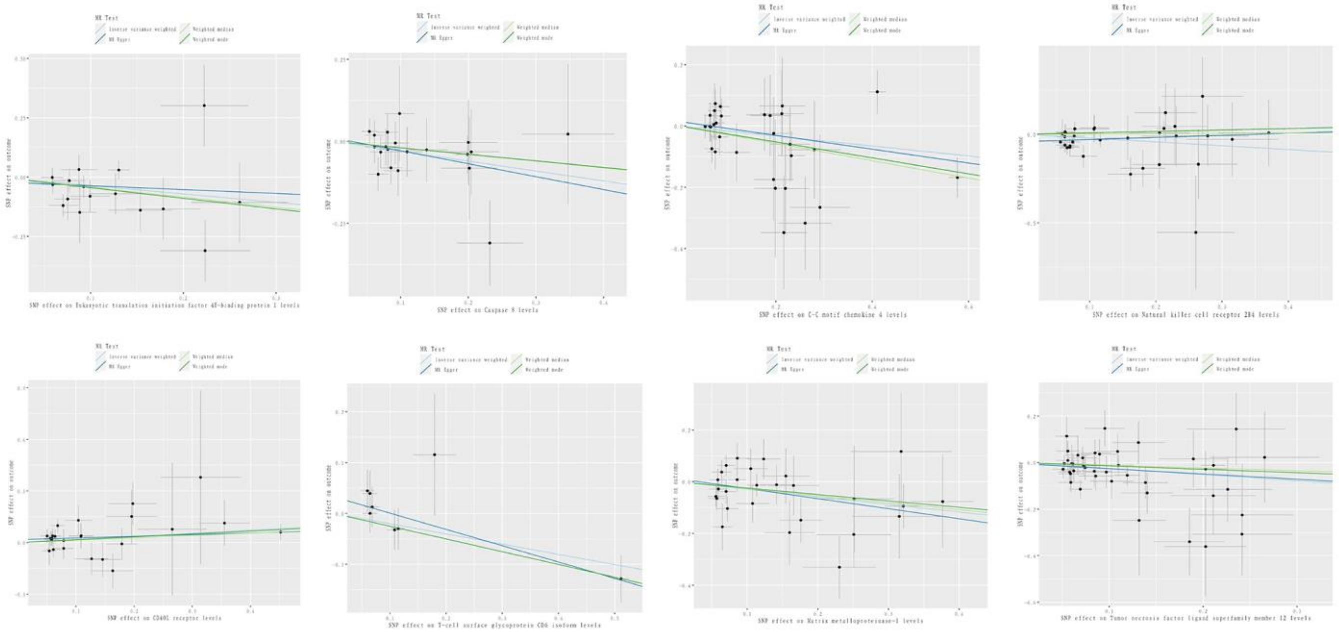

Supplementary Figure 1. The scatter plots for the MR plasma proteins-to-KCa.

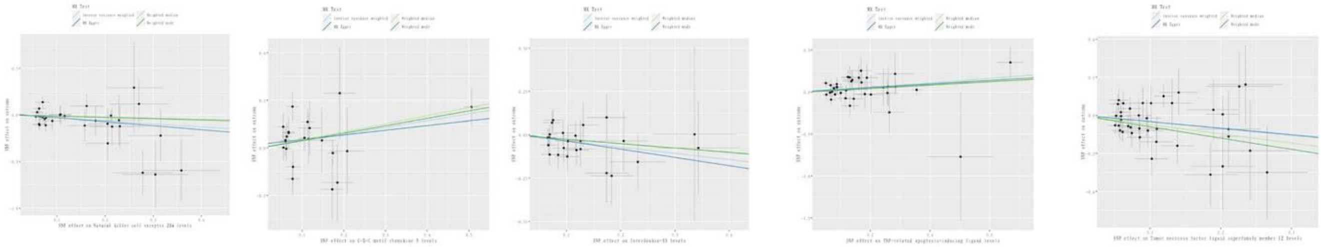

Supplementary Figure 2. The scatter plots for the MR plasma proteins-to-BCa.

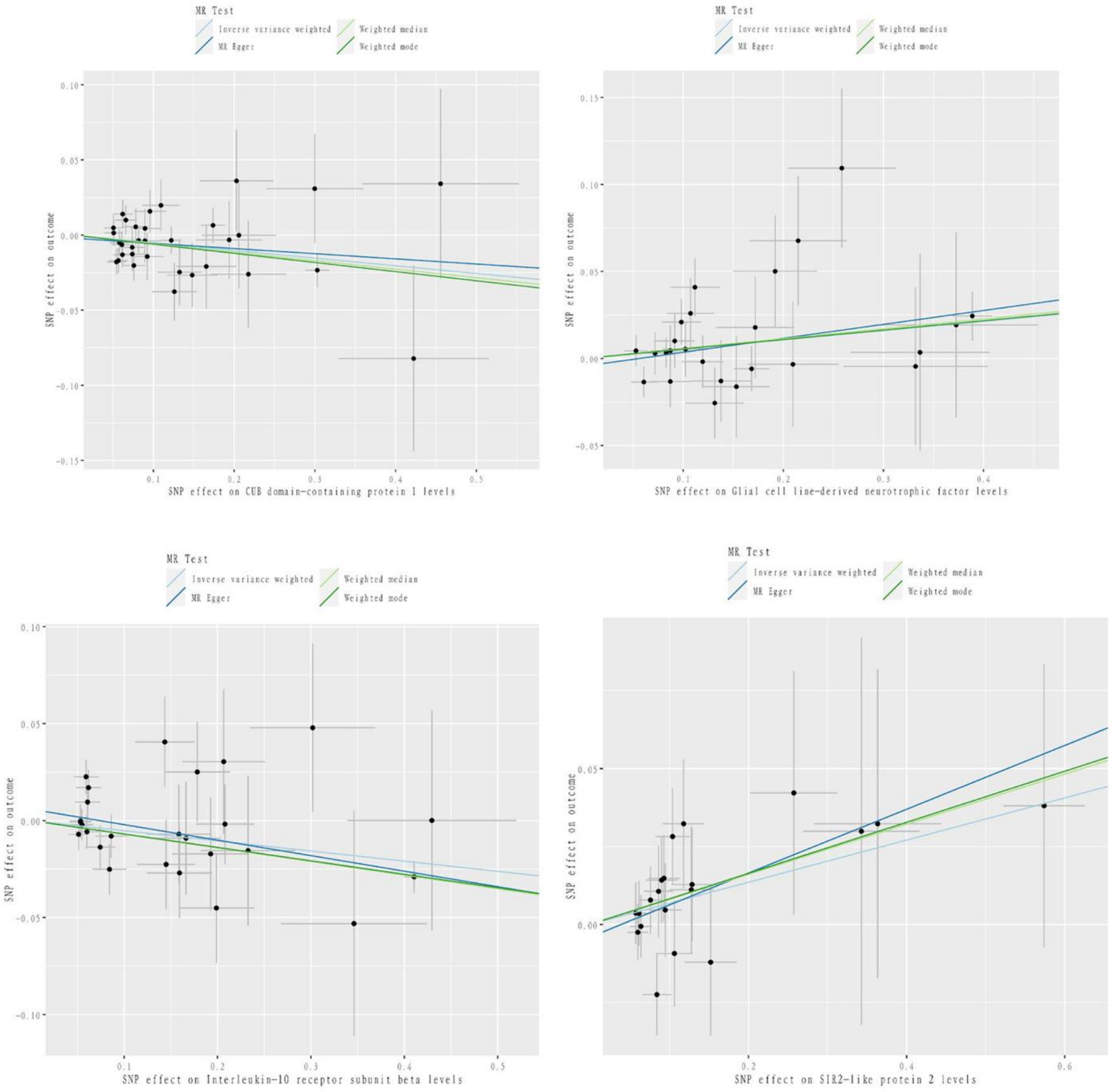

Supplementary Figure 3. The scatter plots for the MR plasma proteins-to-PCa.

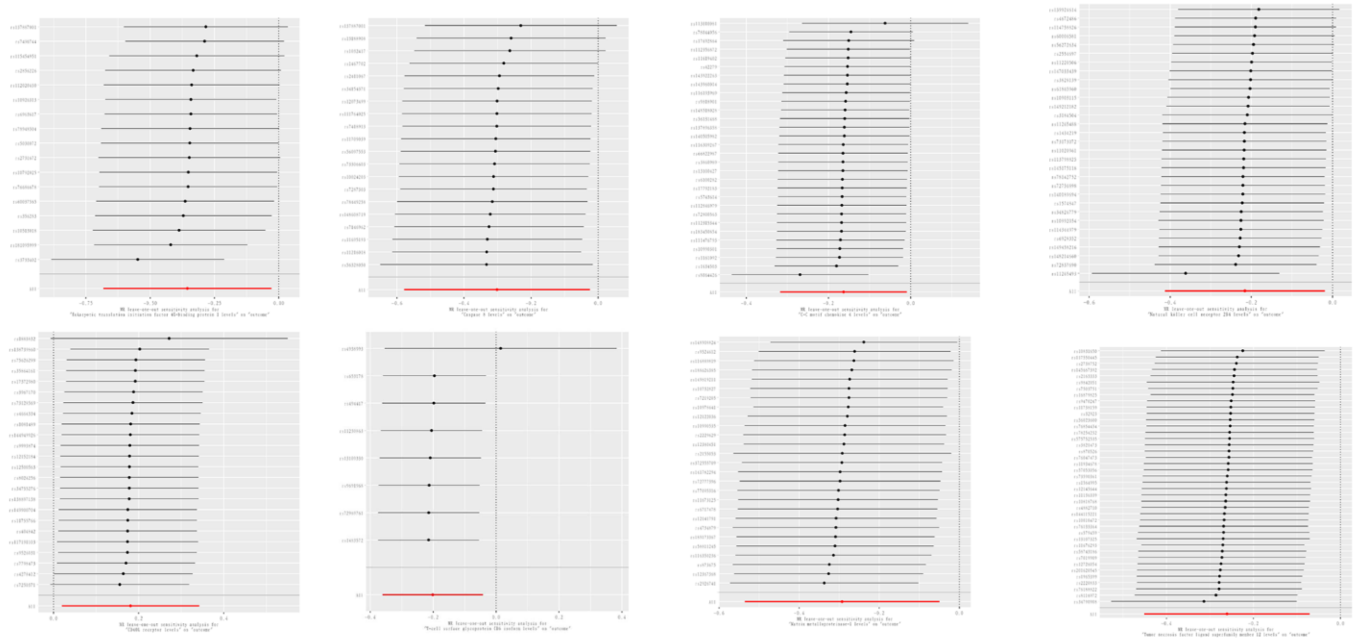

Supplementary Figure 4. The leave-one-out sensitivity analysis between KCa and circulating inflammatory proteins.

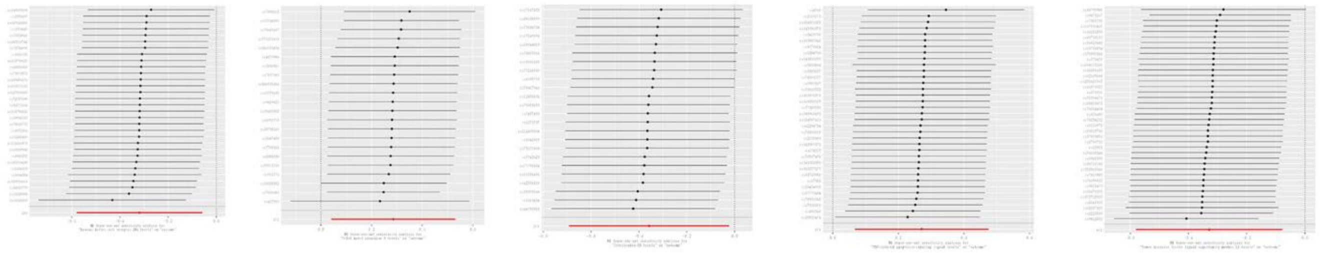

Supplementary Figure 5. The leave-one-out sensitivity analysis between BCa and circulating inflammatory proteins.

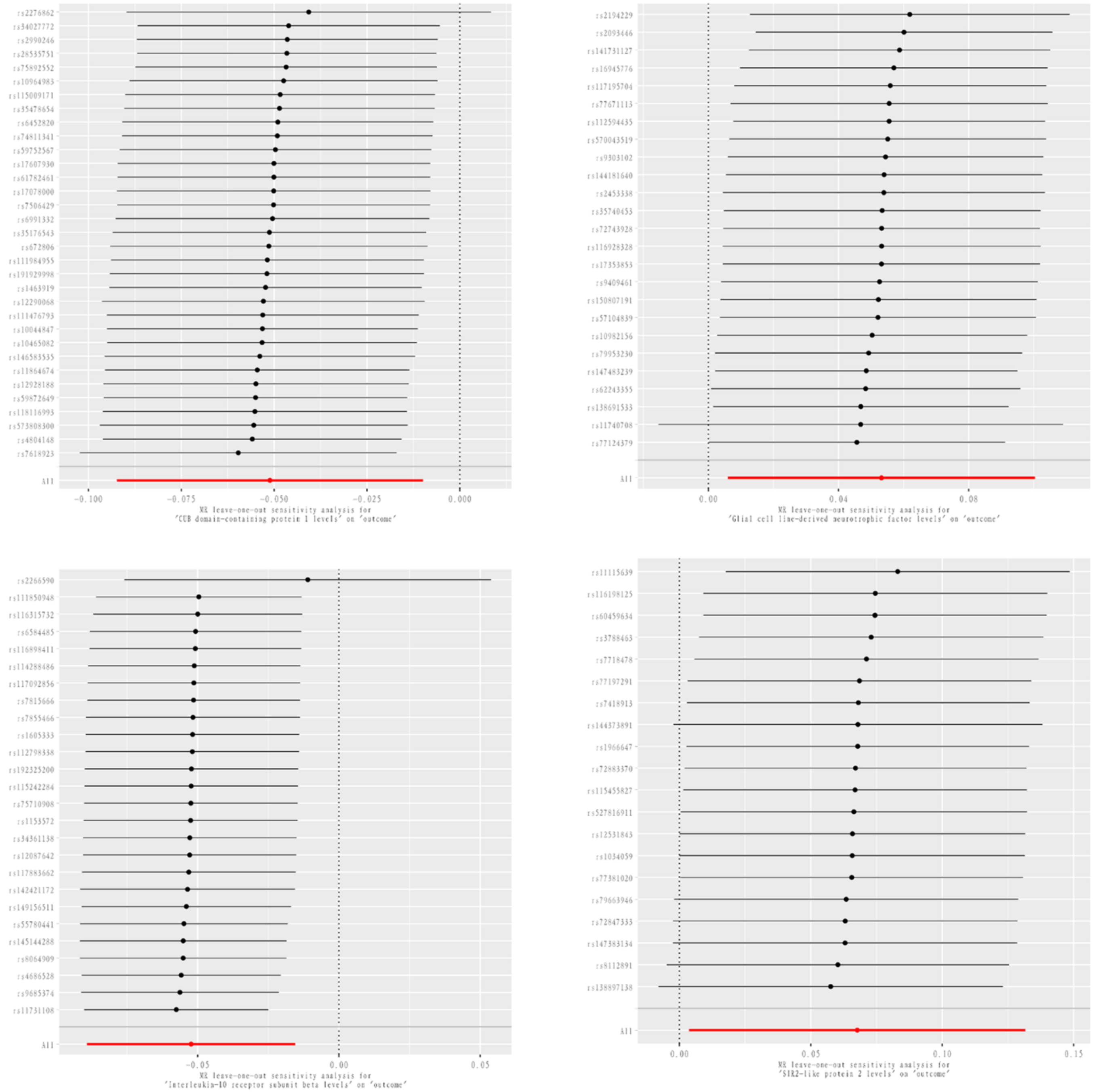

Supplementary Figure 6. The leave-one-out sensitivity analysis between PCa and circulating inflammatory proteins.
